# Hybrid disturbance observer and fuzzy logic controller for a new aerial manipulation system

**DOI:** 10.3389/frobt.2025.1528415

**Published:** 2025-07-07

**Authors:** Alaa Khalifa, Shaaban M. Shaaban, Ahmed Khalifa

**Affiliations:** ^1^ Department of Industrial Electronics and Control Engineering, Faculty of Electronic Engineering, Menoufia University, Menouf, Egypt; ^2^ Center for Scientific Research and Entrepreneurship, Northern Border University, Arar, Saudi Arabia; ^3^ Cardiff School of technologies, Cardiff Metropolitan University, Cardiff, United Kingdom

**Keywords:** aerial manipulation, dynamics, disturbance observer, fuzzy logic controller, kinematics, quadrotor

## Abstract

Aerial manipulation systems are highly attractive for various applications due to their distinctive features. However, the systems discussed in the literature are constrained by either a restricted number of end-effector degrees of freedom (DOFs) or low payload capability. In our previous research, we mounted a manipulator with a gripper on the underside of a quadrotor to enhance environmental interaction. This paper explores a quadrotor equipped with a 2-DOF manipulator featuring a distinctive topology that allows the end-effector to follow a specified 6-DOF trajectory with the least number of actuators required. An overview of the proposed manipulation system, along with its kinematic and dynamic analysis, is presented. Nevertheless, controlling this system presents significant challenges because of its considerable couplings, nonlinearities, and external disturbances. This paper employs a Disturbance Observer (DOb)-based linearization for an aerial manipulation robot. The DOb-based inner loop is responsible for estimating and compensating nonlinearities and disturbances, which simplifies the control problem into a more straightforward linear control algorithm. Subsequently, a fuzzy logic controller is incorporated into the outer loop to achieve the desired control objectives and closed-loop performance while minimizing computational load. Stability analysis of the proposed controller is introduced. Finally, the system is simulated using MATLAB/SIMULINK, and the results demonstrate tracking accuracy during 6-DOF maneuvers under many kinds of disturbances, with low computational load. The system maintains stability during payload exchanges while respecting all actuator constraints (rotor thrust less than 6 N, joint torques less than 0.7 and 0.4 N.m, respectively). These results demonstrate the effectiveness of the proposed control approach. Also, they show that the proposed controller outperforms the DOb-PD controller’s response.

## 1 Introduction

Recently, there has been significant interest in aerial manipulators due to their crucial applications in areas that ground robots cannot reach (Khamseh et al., 2018). Quadrotors, with their exceptional mobility, are employed for mobile manipulation, opening new avenues in robotics (Wei-hong et al., 2021; Xilun et al., 2019). These systems are used for various tasks such as inspection, firefighting, maintenance, delivering light items like mail or quick meals in crowded cities, surveillance, rescue operations, transportation in remote locations, demining, and performing tasks in hazardous environments (Orsag et al., 2018; Li et al., 2024).

Numerous studies have been conducted in the field of aerial manipulation (Ollero et al., 2022; Meng et al., 2020; Ruggiero et al., 2018; Wei-hong et al., 2021). However, existing systems in the literature that utilize a gripper are constrained by the limited degrees of freedom (DOF) of the end-effector. Some systems feature a 2-DOF manipulator, which in some configurations prevents the end-effector from following an arbitrary 6-DOF trajectory. Other systems have a manipulator with more than two DOF, which significantly reduces the system’s payload capacity (Orsag et al., 2017). In Fanni and Khalifa (2017), Khalifa et al. (2016a), Khalifa et al. (2024), Khalifa et al. introduce a novel aerial manipulation system comprising a two-link manipulator with two perpendicular revolute joints. One of the quadrotor’s in-plane axes and the first joint’s axis are parallel to each other. The end-effector can achieve any desired position and orientation thanks to this configuration, which eliminates the need for horizontal movement.

Existing control strategies in the literature for aerial manipulation systems rely on highly complex nonlinear controllers that demand significant computational resources. Achieving stable position holding is a major challenge in aerial manipulation. To accomplish this, a robust control system is necessary to handle disturbances, nonlinearities, uncertainties, and couplings. This robustness challenge was addressed in Khalifa et al. (2016b) by employing a control technique based on DOb, which estimates uncertainties and nonlinear terms. By doing so, the robotic system behaves akin to a multi-SISO linear system, allowing the use of standard linear control methods for designing the outer loop controller and ensuring accurate tracking performance.

However, prior research (Li et al., 2016; Sariyildiz and Ohnishi, 2014; Choi et al., 2014; Tian et al., 2024) related to Disturbance Observer (DOb) techniques faces challenges in estimating system velocity or acceleration, particularly due to limitations in sensors that are available for flying robots. Although it is possible to measure angular velocities and linear accelerations using encoders and an Inertial Measurement Unit (IMU), Tomić and Haddadin (2014) suggests a model-based method to estimate external forces for a basic UAV, relying on IMU data. However, this method requires knowledge of dynamic models, disregards certain nonlinearities and dynamics, employs a nonlinear controller, and exclusively addresses external disturbances without taking system dynamics comprehensively into consideration. As a result, this technique is not well-suited for the complex dynamical and kinematic characteristics of the considered aerial manipulator.

In Tutsoy et al. (2023), a reduced-order Thau observer was presented that focusses on uncertain rotational dynamics and achieves accurate fault detection with just a third-order design. However, it should be expanded to include control signal delays, state measurement delays, data loss, and sensor failures. There are many kinds of modern artificial intelligence-based observers. However, the main issue is balancing computational cost, accuracy, and estimation speed. The aerial robotic manipulation system has very fast dynamics. So, an approach with low computational cost is needed. Also, all of these computations must be accomplished onboard to avoid any delay. On the other hand, the computational time in artificial intelligence-based observers will be long.

To address these limitations, the traditional DOb is adapted to be practical and compatible for the aerial manipulation system proposed in Fanni and Khalifa (2017); Khalifa et al. (2016a), Khalifa et al. (2024); Khalifa and Fanni (2017). In the traditional DOb structure, the DOb estimates system disturbances using velocity or acceleration measurements. However, it is commonly known that the quadrotor’s linear accelerations and angular rates may be obtained straight from the IMU. Furthermore, the angular velocities of the joints may be monitored using an encoder. As a result, we propose a hybrid DOb-based controller for our robotic system. In this hybrid DOb-based control, two distinct DOb loops are employed. The first is based on measured accelerations, and the second on measured velocities. Instead of using differentiation (which makes the system more sensitive to noise) or integration (which causes drift), the proposed scheme uses the measured velocity and acceleration directly from the onboard sensors, with no differentiation or integration. As a result, we propose and implement a hybrid observer that uses raw measurements (both measured velocities and accelerations) in our robust motion control scheme.

Our approach involves several key modifications. First, traditional Dob is redesigned, leveraging angular velocities and linear acceleration data directly obtained from the onboard encoders and IMU. By incorporating these measurements, nonlinearities and disturbances are estimated more effectively. Second, the estimated disturbance data is fed back into the system, enabling compensation. As a result, the system’s behavior becomes linear. Third, in the outer loop, we design a fuzzy logic controller optimized for performance. This controller ensures that the system responds as desired to track 6-DOF trajectories in the task space. Lastly, we construct a simulation environment that includes non-idealities, closely emulating real-world conditions. Through this setup, we validate the effectiveness of our proposed technique. By combining these modifications, we enhance the robustness and feasibility of the DOb for complex aerial manipulators.

This paper is structured as follows: Section 2 provides a description of the robot under consideration. Sections 3, 4 review the kinematics and dynamics analysis of the proposed system, respectively. Section 5 formulates and presents the control system design. Section 6 introduces the simulation results obtained using MATLAB/SIMULINK. Finally, Section 7 highlights the main conclusions.

## 2 Description of the proposed aerial manipulation system

**FIGURE 1:**
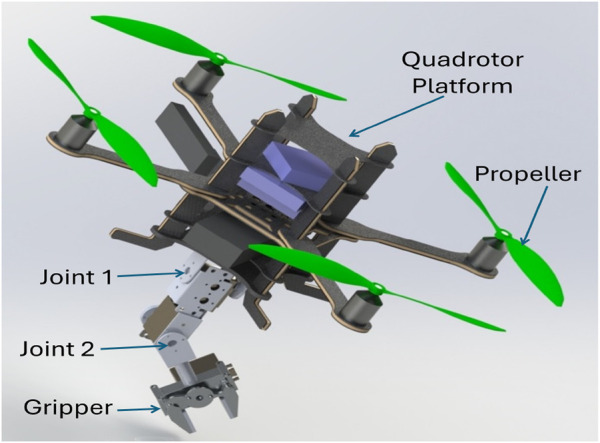
The proposed aerial manipulation system’s 3D CAD model.

**FIGURE 2:**
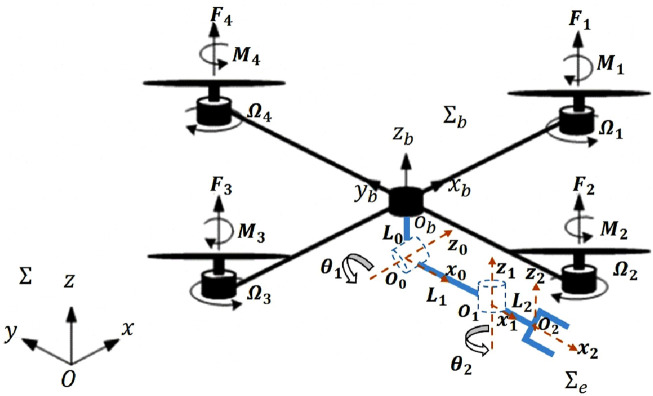
Schematic diagram of the proposed aerial manipulation system with the related frames.

A 3D computer-aided design (CAD) model for the proposed aerial manipulation system is illustrated in Figure 1. The manipulator and the quadrotor itself are the two integral components of this system. Figure 2 offers an illustration that highlights the relevant frames. Notably, these frames define a distinctive topology, enabling the end-effector to achieve arbitrary poses. To maintain consistency, we adhere to the Denavit-Hartenberg (DH) convention for frame transformations (Spong et al., 2020).

Two revolute joints with normal axes are incorporated into the manipulator. Parallel to the quadrotor’s body x -axis is the first revolute joint’s axis $\left(z_{0}\right)$ , which is attached to the body of the quadrotor as shown in Figure 2. The second joint’s axis $\left(z_{1}\right)$ is parallel with the quadrotor’s body y -axis when the manipulator is in its fully extended home position. As a result, the end-effector can now pitch and roll independently of the quadrotor’s horizontal movement. Consequently, this new aerial system enables the manipulation of objects in any position and orientation. This non-redundant system enables the end-effector to achieve full 6-DOF motion using the minimum number of actuators and links, a critical consideration for flight applications. The suggested system sets itself apart from all previously documented systems by offering optimal mobility while maintaining a lightweight design. The increased complexity associated with inverse kinematics and control will be addressed subsequently to demonstrate the end-effector’s ability to accurately follow desired 6-DOF trajectories.

Regarding the quadrotor components, we deliberately select specifications to accommodate a payload of 500 g—exceeding the combined weight of the arm and the maximum payload. Our platform of choice is the Asctec Pelican quadrotor, with each rotor capable of generating a maximum thrust force of 6 N. This thrust capacity is determined through a rigorous identification process. The Asctec Pelican quadrotor incorporates an “asctec Autopilot” Flight Control Unit (FCU) and a modular design that facilitates the integration of various components, including position sensors, computer boards, and the manipulator with its associated avionics. The vehicle’s estimated attitude, magnetic orientation, body accelerations, angular velocities are all provided by the IMU that is a part of the FCU. By combining data from the onboard IMU with either laser/ultrasonic range finder or monocular vision data fusion, the system can estimate the quadrotor’s 6-DOF Achtelik et al. (2011).

The manipulator parts are designed, chosen, bought, and assembled with the goal of weighing no more than 200 g overall. The arm can extend to 22 cm and support a 200 g payload. Three DC motors are used: an HS-5485HB (0.70 N.m maximum torque) for the first joint, and another HS-422 (0.40 N.m maximum torque) for the second joint, an HS-422 (0.40 N.m maximum torque) for the gripper. A Motor Driver (SSC) serves as the intermediary interface between the primary control unit and the motors. Remote control commands for the manipulator’s motors are transmitted wirelessly using a PS2 R/C system. The encoder linked to each joint’s motor provides the angular position and speed of the joint. The interface between the onboard computer and the low-level devices (like PS2 wireless receiver, ultrasonic sensor, and motor driver (SSC)) is accomplished utilizing an Arduino Mega 2,560 board.

## 3 Kinematics analysis

### 3.1 Forward kinematics

Consider the body-fixed reference frame denoted as $\Sigma_{b}$ , $O_{b}$ - $x_{b}\;y_{b}\;z_{b}$ , with its origin located at the quadrotor’s center of mass (as depicted in Figure 2). The position relative to the world-fixed inertial reference frame Σ , O - xyz , represented by the vector $p_{b}={\left[x\;y\;z\right]}^{T}$ . Additionally, $\Phi_{b}$ = ${\left[\psi \;\theta \;\phi \right]}^{T}$ characterizes the orientation of the quadrotor. $R_{b}$ is the rotation matrix that determines this orientation and can be given by

$$R_{b}=\left[\begin{array}{ccc} C_{\psi }C_{\theta } & S_{\phi }S_{\theta }C_{\psi }-S_{\psi }C_{\phi } & S_{\psi }S_{\phi }+C_{\psi }S_{\theta }C_{\phi }\\ S_{\psi }C_{\theta } & C_{\psi }C_{\phi }+S_{\psi }S_{\theta }S_{\phi } & S_{\psi }S_{\theta }C_{\phi }-C_{\psi }S_{\phi }\\ -S_{\theta } & C_{\theta }S_{\phi } & C_{\theta }C_{\phi } \end{array}\right],$$ (1)

where the ZYX yaw-pitch-roll angles represented by the vector $\Phi_{b}$ = ${\left[\psi \;\theta \;\phi \right]}^{T}$ . Note that $S_{*}$ and $C_{*}$ denote abbreviations for sine(∗) and cosine(∗) functions, respectively. Now, we will focus on the frame directly fastened to the manipulator’s end-effector and represented by $\Sigma_{e}$ , $O_{2}$ - $x_{2}\;y_{2}\;z_{2}$ (as depicted in Figure 2). Consequently, the position of $\Sigma_{e}$ relative to the fixed world reference frame Σ can be provided by

$$p_{e}=p_{b}+R_{b}p_{eb}^{b},$$ (2)

where $p_{eb}^{b}$ is the vector, expressed within the reference frame $\Sigma_{b}$ , that denotes $\Sigma_{e}$ ’s position relative to $\Sigma_{b}$ . Additionally, the rotation matrix that can be used to characterize the orientation of $\Sigma_{e}$ is provided by

$$R_{e}=R_{b}R_{e}^{b},$$ (3)

where $\Sigma_{e}$ ’s orientation with respect to $\Sigma_{b}$ is specified by the rotation matrix $R_{e}^{b}$ .

Finding the operational task coordinates $\chi_{e}={\left[x_{e}\;y_{e}\;z_{e}\;\psi_{e}\;\theta_{e}\;\phi_{e}\right]}^{T}$ based on the coordinates of the joint or vehicle space, denoted as $q={\left[x\;y\;z\;\psi \;\theta \;\phi \;\theta_{1}\;\theta_{2}\right]}^{T}$ , is the challenge of forward kinematics. Eight variables, q , make up the input for the forward kinematics, while six variables, $\chi_{e}$ , are generated from a series of six algebraic equations to make up the output. Equation 2 may be used to determine the end-effector’s position. Furthermore, it is possible to get the end-effector’s Euler angles, $\Phi_{e}$ , from $R_{e}$ as illustrated in Equation 3.

### 3.2 Inverse kinematics

Finding the coordinates of the joint or vehicle space, denoted as q based on the operational task coordinates $\chi_{e}$ , is the challenge of inverse kinematics. The robot’s control depends on the inverse kinematics solution, which makes it possible to determine the quadrotor’s required movements and the manipulator joints’ angles in order to position the end effector at a specified location and orientation. The end effector’s rotations can be described using various methods, one of which is the Euler angles Slabaugh (1999). $T_{2}^{I}$ is the overall transformation matrix that connects the inertial world frame and the end effector frame. It is defined by

$$T_{2}^{I}=A_{B}^{I}A_{0}^{B}A_{1}^{0}A_{2}^{1}$$ (4)

As a function of the end effector variables $\chi_{e}$ , specify this transformation matrix’s general format as shown below

$$T_{ee}=\left[\begin{array}{cccc} r_{11} & r_{12} & r_{13} & x_{e}\\ r_{21} & r_{22} & r_{23} & y_{e}\\ r_{31} & r_{32} & r_{33} & z_{e}\\ 0 & 0 & 0 & 1 \end{array}\right]$$ (5)

Since the objective is to get the inverse kinematics for the reset position, $T_{2}^{I}$ in Equation 4 may be rewritten by substituting ϕ=θ=0 as follows

$$T_{2}^{I}=\left[\begin{array}{cccc} C_{\psi }S_{\theta_{2}}+C_{\theta_{1}}C_{\theta_{2}}S_{\psi } & C_{\psi }C_{\theta_{2}}-C_{\theta_{1}}S_{\psi }S_{\theta_{2}} & S_{\psi }S_{\theta_{1}} & X+L_{1}C_{\theta_{1}}S_{\psi }+L_{2}C_{\psi }S_{\theta_{2}}+L_{2}C_{\theta_{1}}C_{\theta_{2}}S_{\psi }\\ S_{\psi }S_{\theta_{2}}-C_{\psi }C_{\theta_{1}}C_{\theta_{2}} & C_{\theta_{2}}S_{\psi }+C_{\psi }C_{\theta_{1}}S_{\theta_{2}} & -C_{\psi }S_{\theta_{1}} & Y-L_{1}C_{\psi }C_{\theta_{1}}+L_{2}S_{\psi }S_{\theta_{2}}-L_{2}C_{\psi }C_{\theta_{1}}C_{\theta_{2}}\\ -C_{\theta_{2}}S_{\theta_{1}} & S_{\theta_{1}}S_{\theta_{2}} & C_{\theta_{1}} & Z-L_{0}-L_{1}S_{\theta_{1}}-L_{2}C_{\theta_{2}}S_{\theta_{1}}\\ 0 & 0 & 0 & 1 \end{array}\right]$$ (6)

Using this equation, we can deduce the inverse kinematics for the system. Based on the formulation in Equation 6, the process begins with determining the inverse orientation, which is then succeeded by the calculation of inverse position. The inverse orientation encompasses three cases, outlined as follows.

CASE 1 Assume that neither $r_{13}$ nor $r_{23}$ is equal to zero. From Equation 6, we conclude that $sin\left(\theta_{1}\right)$ is not equal to zero and that $r_{33}$ is not equal to ±1 . Therefore, it follows that $cos\left(\theta_{1}\right)=r_{33}$ and $sin\left(\theta_{1}\right)=\pm \sqrt{1-r_{33}^{2}}$ , leading to the conclusion

$$\theta_{1}=atan2\left(\pm \sqrt{1-r_{33}^{2}},r_{33}\right)$$ (7)

$$\psi =atan2\left(\pm r_{13},\mp r_{23}\right)$$ (8)

$$\theta_{2}=atan2\left(\pm r_{32},\mp r_{31}\right)$$ (9)

Consequently, there are two possible solutions based on the selected sign for $sin\left(\theta_{1}\right)$ . In cases where $r_{13}$ and $r_{23}$ equal zero, the orthogonality of $T_{ee}$ indicates that $r_{33}$ must be either +1 or −1 .

CASE 2 When $r_{13}$ = $r_{23}=0$ and $r_{33}=1$ , it follows that $\cos\left(\theta_{1}\right)$ equals 1 and $\sin\left(\theta_{1}\right)$ equals 0, leading to $\theta_{1}$ being 0. In this scenario, based on the rotation matrix from Equation 6, the expression for $\theta_{2}+\psi$ can be calculated as

$$\theta_{2}+\psi =atan2\left(r_{11},r_{12}\right)$$ (10)

We can assign any value to ψ to determine $\theta_{2}$ , resulting in an infinite number of solutions.

CASE 3 When $r_{13}=r_{23}=0$ and $r_{33}=-1$ , it follows that $\cos\left(\theta_{1}\right)=-1$ and $\sin\left(\theta_{1}\right)=0$ , leading to $\theta_{1}=\pi$ . In this scenario, from Equation 6, $\theta_{2}-\psi$ can be calculated using the equation

$$\theta_{2}-\psi =atan2\left(r_{11},r_{12}\right)$$ (11)

Any value can be assigned to ψ to find $\theta_{2}$ , resulting in an infinite number of potential solutions.

In both cases two and three, one might set ψ=0 to determine $\theta_{2}$ . Ultimately, the inverse position can be established through

$$X=x_{e}-\left(L_{1}C_{\theta_{1}}S_{\psi }+L_{2}C_{\psi }S_{\theta_{2}}+L_{2}C_{\theta_{1}}C_{\theta_{2}}S_{\psi }\right)$$ (12)

$$Y=y_{e}-\left(-L_{1}C_{\psi }C_{\theta_{1}}+L_{2}S_{\psi }S_{\theta_{2}}-L_{2}C_{\psi }C_{\theta_{1}}C_{\theta_{2}}\right)$$ (13)

$$Z=z_{e}-\left(-L_{0}-L_{1}S_{\theta_{1}}-L_{2}C_{\theta_{2}}S_{\theta_{1}}\right)$$ (14)

## 4 Dynamics analysis

**FIGURE 3:**
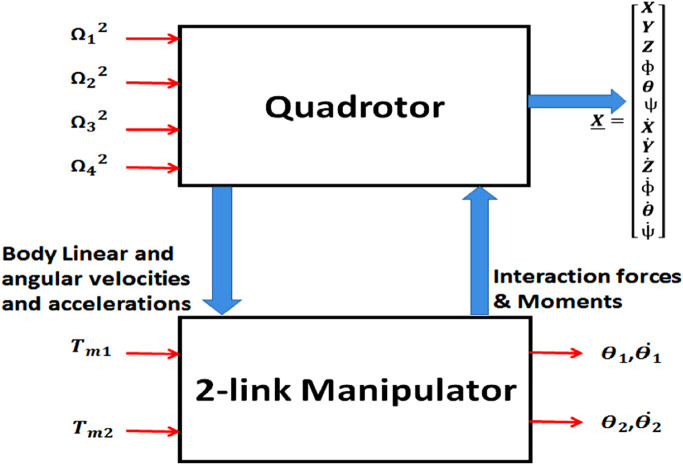
Effects of integrating a manipulator with a quadrotor.

Figure 3 presents a schematic diagram showing the effects of integrating a manipulator with a quadrotor. To analyze the dynamics of the manipulator, the Recursive Newton-Euler method (Tsai, 1999; Fanni and Khalifa, 2017) is employed to formulate the equations governing motion. Given that the quadrotor serves as the manipulator’s base platform, the initial angular and linear accelerations and velocities utilized in the Newton-Euler method are those of the quadrotor represented in its body frame. By considering the link (of length $L_{0}$ ) that is attached to the quadrotor as the base link and implementing the Newton-Euler technique for the manipulator, one can derive the equations of motion for the manipulator. Also, the forces and moments generated by the manipulator that influence the quadrotor can be obtained.

The quadrotor platform is considered a rigid and symmetrical body. Similarly, each manipulator link is assumed to be rigid. The dynamic behavior of the manipulator is described by

$$M_{1}\left(q\right){\ddot{\theta }}_{1}+N_{1}\left(q,\dot{q},\ddot{q}\right)=\tau_{m_{1}},$$ (15)

$$M_{2}\left(q\right){\ddot{\theta }}_{2}+N_{2}\left(q,\dot{q},\ddot{q}\right)=\tau_{m_{2}},$$ (16)

where $\tau_{m_{1}}$ and $\tau_{m_{2}}$ represent the torques generated by the manipulator’s actuators. The terms $M_{1}\left(q\right)$ , $M_{2}\left(q\right)$ , $N_{1}\left(q,\dot{q},\ddot{q}\right)$ , and $N_{2}\left(q,\dot{q},\ddot{q}\right)$ introduce nonlinearities into the system and are functions of the system’s states ( q , $\dot{q}$ ), and accelerations $\ddot{\chi_{b}}$ . The dynamic behavior of the quadrotor, incorporating the force and torque contributions from the manipulator, was determined using the Newton-Euler formulation. These equations are expressed as follows

$$m\ddot{x}=T\left(C_{\psi }S_{\theta }C_{\phi }+S_{\psi }S_{\phi }\right)+F_{m,q_{x}}$$ (17)

$$m\ddot{y}=T\left(S_{\psi }S_{\theta }C_{\phi }-C_{\psi }S_{\phi }\right)+F_{m,q_{y}}$$ (18)

$$m\ddot{z}=-mg+TC_{\theta }C_{\phi }+F_{m,q_{z}}$$ (19)

$$I_{x}\ddot{\phi }=\dot{\theta }\dot{\phi }\left(I_{y}-I_{z}\right)-I_{r}\dot{\theta }\bar{\Omega }+T_{a_{1}}+M_{m,q_{\phi }}^{b}$$ (20)

$$I_{y}\ddot{\theta }=\dot{\psi }\dot{\phi }\left(I_{z}-I_{x}\right)+I_{r}\dot{\phi }\bar{\Omega }+T_{a_{2}}+M_{m,q_{\theta }}^{b}$$ (21)

$$I_{z}\ddot{\psi }=\dot{\theta }\dot{\phi }\left(I_{x}-I_{y}\right)+T_{a_{3}}+M_{m,q_{\psi }}^{b}$$ (22)

where $F_{m,q_{x}}$ , $F_{m,q_{y}}$ , and $F_{m,q_{z}}$ indicate the forces applied by the manipulator on the quadrotor along the x , y , and z axes in the inertial frame, respectively. Likewise, $M_{m,q_{\phi }}^{b}$ , $M_{m,q_{\theta }}^{b}$ , and $M_{m,q_{\psi }}^{b}$ refer to the moments produced by the manipulator around the $x_{b}$ , $y_{b}$ , and $z_{b}$ axes of the quadrotor’s body frame.

The parameters in Equations 17–22 are described as follow. The quadrotor’s total mass is denoted by m . Each rotor j possesses an angular velocity $\Omega_{j}$ . As a result, it generates both thrust force $F_{j}$ and drag moment $M_{j}$ , which can be expressed as follows

$$F_{j}=K_{f_{j}}\Omega_{j}^{2},$$ (23)

$$M_{j}=K_{m_{j}}\Omega_{j}^{2},$$ (24)

where $K_{f_{j}}$ and $K_{m_{j}}$ correspond to the thrust and drag coefficients for rotor j , respectively.

The four rotors’ combined thrust is symbolized by T and provided by

$$T=\overset{4}{\underset{j=1}{\sum }}\left(F_{j}\right).$$ (25)

The control torques around the quadrotor’s body axes $x_{b}$ , $y_{b}$ , and $z_{b}$ are indicated by $T_{a_{1}}$ , $T_{a_{2}}$ , and $T_{a_{3}}$ , respectively. They are provided by

$$T_{a_{1}}=d\left(F_{4}-F_{2}\right),$$ (26)

$$T_{a_{2}}=d\left(F_{3}-F_{1}\right),$$ (27)

$$T_{a_{3}}=-M_{1}+M_{2}-M_{3}+M_{4}.$$ (28)

d is the perpendicular distance between each rotor’s rotation axis and the centre of mass of the quadrotor. The rotor speed vector $\bar{\Omega }$ is defined as follows:

$$\bar{\Omega }=\Omega_{1}-\Omega_{2}+\Omega_{3}-\Omega_{4}.$$ (29)

The rotor’s inertia is represented by $I_{r}$ . Under the assumption that the vehicle exhibits symmetry along the $x_{b}$ , $y_{b}$ , and $z_{b}$ axes, the inertia matrix of the quadrotor relative to its body frame is denoted by $I_{f}$ . The mathematical depiction of the aerial manipulation system’s dynamic model is outlined as

$$M\left(q\right)\ddot{q}+C\left(q,\dot{q}\right)\dot{q}+G\left(q\right)+d_{ex}=Bu,$$ (30)

The matrix M , an 8×8 symmetric positive definite matrix, encapsulates the inertia characteristics of the combined system. The Coriolis and centrifugal effects are represented by the matrix $C\;\in R^{8\times 8}$ , while the gravitational forces are captured in the 8-dimensional vector G . External disturbances acting on the system are aggregated in the vector $d_{ex}\;\in R^{8}$ . The actuator inputs are organized into the 6-dimensional control vector $u={\left[F_{1}\;F_{2}\;F_{3}\;F_{4}\;\tau_{m_{1}}\;\tau_{m_{2}}\right]}^{T}\;\in R^{6}$ . The input matrix $B=H\left(q\right)\;N\left(K_{f_{j}},K_{m_{j}},d\right)$ maps the actuator inputs to the generated body forces and moments. The control matrix N has the following structure

$$N=\left[\begin{array}{cccccc} 0 & 0 & 0 & 0 & 0 & 0\\ 0 & 0 & 0 & 0 & 0 & 0\\ 1 & 1 & 1 & 1 & 0 & 0\\ \gamma_{1} & -\gamma_{2} & \gamma_{3} & -\gamma_{4} & 0 & 0\\ -d & 0 & d & 0 & 0 & 0\\ 0 & -d & 0 & d & 0 & 0\\ 0 & 0 & 0 & 0 & 1 & 0\\ 0 & 0 & 0 & 0 & 0 & 1 \end{array}\right],$$ (31)

where $\gamma_{j}=K_{m_{j}}/K_{f_{j}}$ . Also, the body input forces are converted to be represented in Σ by the matrix $H\;\in R^{8\times 8}$ , as follows

$$H=\left[\begin{array}{ccc} R_{b} & O_{3} & O_{2}\\ O_{3} & T_{b}^{T}R_{b} & O_{2}\\ O_{2\times 3} & O_{2\times 3} & I_{2} \end{array}\right].$$ (32)

By analyzing the translational dynamic part of Equations 17–19, the following second-order nonholonomic constraint equations can be derived

$$\sin\left(\phi \right)-\frac{{\ddot{x}}_{f}S_{\psi }-{\ddot{y}}_{f}C_{\psi }}{\sqrt{{\ddot{x}}_{f}^{2}+{\ddot{y}}_{f}^{2}+{\ddot{z}}_{f}^{2}}}=0,$$ (33)

$$\tan\left(\theta \right)-\frac{{\ddot{x}}_{f}C_{\psi }+{\ddot{y}}_{f}S_{\psi }}{{\ddot{z}}_{f}}=0,$$ (34)

where ${\ddot{x}}_{f}$ = $\ddot{x}-\frac{F_{m,q_{x}}}{m}$ , ${\ddot{y}}_{f}$ = $\ddot{y}-\frac{F_{m,q_{y}}}{m}$ , and ${\ddot{z}}_{f}$ = $\ddot{z}+g-\frac{F_{m,q_{z}}}{m}$ .

It is important to highlight that the force terms in the equations mentioned previously are function of the states of the system and their derivatives. By substituting the desired trajectories of the other variables into Equations 33, 34, the desired trajectories for ϕ and θ can be determined.

It is worth mentioning that the dynamics of the actuators are much faster than the dynamics of the aerial manipulation system. So, they can be neglected.

Wind dynamics, $\tau_{w}$ , may be viewed as external disturbances, hence it is inherently included in the $d_{ex}$ term. Wind dynamics, $\tau_{w}$ , may be modelled as follows Hsu (2011), Viktor et al. (2015):

Average wind speed is computed by

$$V_{wz}=V_{w_{z_{0}}}\frac{z}{z_{0}},$$ (35)

where $V_{wz}$ represents the wind speed at altitude z , whereas $V_{w_{z_{0}}}$ represents the recorded wind speed at altitude $z_{0}$ .

To replicate wind disturbances, calculate the wind force, $F_{w}$ , which affects the platform rather than the wind speed. The force may be calculated using

$$F_{w}=0.61*A_{e}V_{wz}^{2},$$ (36)

where 0.61 is used to convert the wind speed to pressure, and $A_{e}$ is the quadrotor’s impact effective area, which is determined by its construction and orientation. This force may be projected on the frame Σ as

$$\begin{array}{c} F_{wx}=f_{1}\;z^{2}sin\left(\theta \right)+f_{2}\;z^{2}cos\left(\theta \right),\\ F_{wy}=f_{3}\;z^{2}sin\left(\phi \right)+f_{4}\;z^{2}cos\left(\phi \right), \end{array}$$ (37)

where $f_{1}=0.61*A_{e_{1}}{\left(\frac{V_{w_{z_{0}}}}{z_{0}}\right)}^{2}cos\left(\psi_{w}\right)$ , $f_{2}=0.61*A_{e_{2}}{\left(\frac{V_{w_{z_{0}}}}{z_{0}}\right)}^{2}cos\left(\psi_{w}\right)$ , $f_{3}=0.61*A_{e_{1}}{\left(\frac{V_{w_{z_{0}}}}{z_{0}}\right)}^{2}sin\left(\psi_{w}\right)$ , $f_{4}=0.61*A_{e_{2}}{\left(\frac{V_{w_{z_{0}}}}{z_{0}}\right)}^{2}sin\left(\psi_{w}\right)$ , $\psi_{w}$ denotes the wind direction angle, whereas $A_{e_{1}}$ and $A_{e_{2}}$ are determined by the quadrotor’s design parameters.

## 5 Controller design

We wish to accomplish the following objectives by designing the control input τ :

1. System Linearization: The external disturbances and nonlinearities of the system are estimated by utilizing measurement data directly obtained from onboard sensors. This guarantees that the error in estimation, ${\tilde{\tau }}^{\mathrm{dis}}=\tau^{\mathrm{dis}}-{\hat{\tau }}^{\mathrm{dis}}$ converges to zero as time progresses to infinity.
2. Robust Stability: In spite of uncertainties, external disturbances, and measurement noise, the robotic manipulation system maintains stability and robustness.
3. Trajectory Tracking The deviation in the position of the end-effector tends to diminish to zero as time progresses towards infinity.

Our suggestion for a control strategy is to use a modified DOb and fuzzy logic controller to meet these control objectives. In this approach, system uncertainties, nonlinearities, and external disturbances $\left(\tau^{\mathrm{dis}}\right)$ are regarded as disturbances. These disturbances are estimated using angular velocity and linear acceleration measurements $\left({\hat{\tau }}^{\mathrm{dis}}\right)$ and are eliminated by the DOb. This allows the system to be viewed as linear Single Input Single Output (SISO) plants. Consequently, the outer loop employs a fuzzy logic controller to generate $\tau^{\mathrm{des}}$ in order to acquire the required system performance.

### 5.1 DOb loop

**FIGURE 4:**
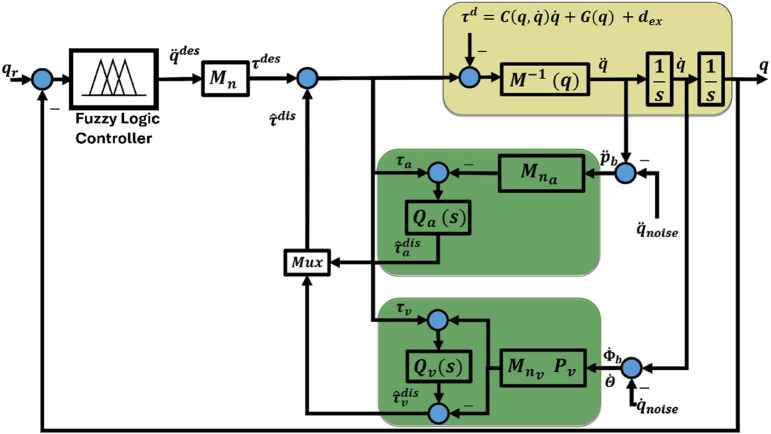
Block diagram of the proposed DOb and fuzzy logic controller.

Figure 4 illustrates a block diagram for the DOb controller in the inner loop, which will later facilitate the creation of a robust control system for the intended aerial manipulation system. It is widely known that the IMU can directly capture the quadrotor’s angular rates and linear accelerations. Moreover, joint angular velocities can be obtained using an encoder. Consequently, two separate DOb loops are utilized, one utilizing the measured velocity and the other utilizing the detected acceleration.

In Figure 4, the expression $M_{n}=\left[\begin{array}{cc} M_{n_{a}} & O_{3\times 5}\\ O_{5\times 3} & \;M_{n_{v}} \end{array}\right]\;\in R^{8\times 8}$ denotes the nominal inertia matrix of the system. Here, $M_{n_{a}}\;\in R^{3\times 3}$ represents the nominal inertia associated with accelerations ${\ddot{p}}_{b}$ , whereas $M_{n_{v}}\;\in R^{5\times 5}$ corresponds to the nominal inertia related to velocities ${\dot{\Phi }}_{b}$ and $\dot{\Theta }$ . The variables τ and $\tau^{\mathrm{des}}$ indicate the current and desired inputs to the robotic system, respectively. The matrix $Q\left(s\right)=diag\left(\left[\frac{g_{1}}{s+g_{1}}\;\ldots \frac{g_{i}}{s+g_{i}}\;\ldots \frac{g_{8}}{s+g_{8}}\right]\right)\;\in R^{8\times 8}$ functions as the low-pass filter matrix for the DOb, with $Q_{a}\left(s\right)=diag\left(\left[\frac{g_{1}}{s+g_{1}}\;\ldots \frac{g_{3}}{s+g_{3}}\right]\right)$ , and $Q_{v}\left(s\right)=diag\left(\left[\frac{g_{4}}{s+g_{4}}\;\ldots \frac{g_{8}}{s+g_{8}}\right]\right)$ . The matrix $P=diag\left(\left[g_{1}\;\ldots g_{i}\;\ldots g_{8}\right]\right)$ indicates the bandwidth for the $i^{th}$ variable of q , while $P_{v}=diag\left(\left[g_{4}\;\ldots g_{i}\;\ldots g_{8}\right]\right)$ pertains to the portion related to velocity. Additionally, the term $\tau^{\mathrm{dis}}$ reflects the disturbances affecting the system, encompassing Coriolis, centrifugal, and gravitational influences, while ${\hat{\tau }}^{\mathrm{dis}}={\left[{\hat{\tau }}_{a}^{{dis}^{T}}\;{\hat{\tau }}_{v}^{{dis}^{T}}\right]}^{T}$ denotes the estimated disturbances within the system.

The system disturbance, $\tau^{\mathrm{dis}}$ , can be considered as

$$\begin{array}{c} \tau^{\mathrm{dis}}=\left(M\left(q\right)-M_{n}\right)\ddot{q}+\tau^{d},\\ \tau^{d}=C\left(q,\dot{q}\right)\dot{q}+G\left(q\right)+d_{ex}. \end{array}$$ (38)

The control input, τ , shown in Figure 4 can be determined as

$$\tau =M_{n}{\ddot{q}}^{\mathrm{des}}+{\hat{\tau }}^{\mathrm{dis}},$$ (39)

where

$${\hat{\tau }}^{\mathrm{dis}}=Q\left(\tau -M_{n}\ddot{q}\right).$$ (40)

When the DOb operates flawlessly, it can be presumed that all external and internal disturbances are accurately estimated and mitigated (i.e., ${\hat{\tau }}^{\mathrm{dis}}=\tau^{\mathrm{dis}}$ ). As a result, the relationship between the input to the DOb loop $\left(\tau^{\mathrm{des}}\right)$ and the output of the robotic manipulator is described as

$$M_{n}\ddot{q}=\tau^{\mathrm{des}}.$$ (41)

Given $M_{n}$ is a diagonal matrix, the system can be considered as multi-decoupled linear SISO systems, as

$$M_{n_{ii}}{\ddot{q}}_{i}=\tau_{i}^{\mathrm{des}},$$ (42)

or more straightforwardly in the acceleration space as

$${\ddot{q}}_{i}={\ddot{q}}_{i}^{\mathrm{des}}.$$ (43)

The final step in designing the DOb-based controller is to develop the tracking controller in the DOb outer loop. A fuzzy logic controller for the system described in Equation 43 is selected.

### 5.2 Fuzzy logic controller

In recent times, fuzzy logic control has emerged as a viable substitute for traditional control algorithms in controlling complex processes (Sridharan, 2022; Rzayev et al., 2023; Baharuddin and Basri, 2023; Lara Alabazares et al., 2021; Hosseinpour and Martynenko, 2022). It offers an effective approach for developing controllers by utilizing heuristic data, making it suitable for various challenging control applications. Additionally, it combines the benefits of conventional controllers with the expertise of human operators. This paper introduces the design of an intelligent controller for an aerial manipulation system, utilizing MATLAB simulation (Simulink).

A fuzzy logic controller is composed of three main components: the Fuzzification module, Inference Engine and Rule Base, and Defuzzification module (Siddique and Siddique, 2014). The process of obtaining a set of fuzzy membership values from a crisp input value is referred to as fuzzification. To facilitate a seamless mapping of the system, the membership functions should have some degree of overlap. Fuzzy rules, crucial for representing knowledge and past experiences in fuzzy logic, are expressed as conditional statements of the form: If <condition> , Then <action> .

The inference engine involves assessing fuzzy rules to generate an output corresponding to each rule (Kovacic and Bogdan, 2018; Domingos et al., 2016). The output from the fuzzification module, which reflects the degree of membership functions of the input fuzzy sets pertaining to the current state of the process, is compared against each rule’s antecedent to determine a match degree for every rule. This match degree influences the adjustment of the control output variable specified in the rule’s consequent. Composition refers to the integration of the results from all the rules. The result of the combination process is a clipped fuzzy set that signifies the fuzzy values of the control output variable. Following the fuzzy reasoning, we obtain a linguistic output variable that must be converted into a precise crisp value. The goal is to obtain a singular crisp numeric value that most accurately reflects the inferred fuzzy values of the linguistic output variable. Defuzzification effectively translates the output from the fuzzy domain back into the crisp domain.

In Figure 4, fuzzy logic controllers have been developed to manage the position of every joint in the aerial manipulation system. These controllers utilize a similar set of inputs, which include the error e , the difference between the target and actual joint position, and the rate of change of this error de .

**FIGURE 5:**
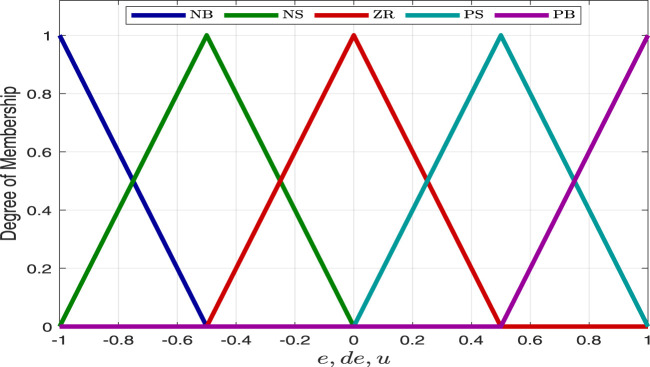
Membership functions of the fuzzy logic controllers.

Each fuzzy logic controller employs five symmetric triangular membership functions with linguistic labels of Negative Big NB , Negative Small NS , Zero ZR , Positive Small PS , and Positive Big PB to represent input and output values. These membership functions are overlapped as shown in Figure 5. To achieve the desired performance, the scaling factors for the error $K_{ei}$ , change of error $K_{\mathrm{dei}}$ , and fuzzy output $K_{ui}$ of each FLC were carefully tuned.

The fuzzy logic controllers share an identical rule base, provided in Table 1, crafted to function as a PD-like fuzzy logic controller. The Mamdani fuzzy inference approach is utilized, employing a min-max operator for aggregation and the center of gravity technique for defuzzification.

**TABLE 1:** Rule base of the fuzzy logic controllers.

*e*	*de*				
	*NB*	*NS*	*ZR*	*PS*	*PB*
NB	NB	NB	NB	NS	ZR
NS	NB	NB	NS	ZR	PS
ZR	NB	NS	ZR	PS	PB
PS	NS	ZR	PS	PB	PB
PB	ZR	PS	PB	PB	PB

### 5.3 Stability analysis

Control input, τ , is provided as

$$\begin{array}{c} \tau =\frac{1}{\left(1-Q\left(s\right)\right)}\left[M_{n}{\ddot{q}}^{\mathrm{des}}-Q\left(s\right)M_{n}\ddot{q}\right]=M_{n}{\ddot{q}}^{\mathrm{des}}+M_{n}Pe_{v},e_{v}={\dot{q}}^{\mathrm{des}}-\dot{q}. \end{array}$$ (44)

The application of this control law leads to

$$\begin{array}{c} M\left(q\right){\dot{e}}_{v}+C\left(q,\dot{q}\right)e_{v}+K_{v}e_{v}=\delta ,\;K_{v}=PM_{n}, \end{array}$$ (45)

where

$$\begin{array}{c} \delta =\Delta M\left(q\right){\ddot{q}}^{\mathrm{des}}+C\left(q,\dot{q}\right){\dot{q}}^{\mathrm{des}}+G\left(q\right)+d_{ex},\;\Delta M\left(q\right)=M\left(q\right)-M_{n}. \end{array}$$ (46)

The following may be used to establish the stability of the inner loop:

Consider a Lyapunov function as

$$V=\frac{1}{2}e_{v}^{T}M\left(q\right)e_{v}.$$ (47)

This function has the following time derivative

$$\dot{V}=e_{v}^{T}M\left(q\right){\dot{e}}_{v}+\frac{1}{2}e_{v}^{T}\dot{M}\left(q\right)e_{v}.$$ (48)

When Equation 48 is substituted with Equation 45,

$$\dot{V}=e_{v}^{T}\delta -e_{v}^{T}K_{v}e_{v}+\frac{1}{2}e_{v}^{T}\left(\dot{M}\left(q\right)-2C\left(q,\dot{q}\right)\right)e_{v}.$$ (49)

This proof will use the dynamic equation of motion’s properties Equation 30. These properties are From et al. (2014), Spong et al. (2020):

property 1

$$\lambda_{\min}\|{\nu \|}^{2}\leq \nu^{T}M\left(q\right)\nu \leq \lambda_{\max}\|{\nu \|}^{2},$$ (50)

property 2

$$\nu^{T}\left(\dot{M}\left(q\right)-2C\left(q,\dot{q}\right)\right)\nu =0,$$ (51)

where $\nu \in R^{8}$ denotes an 8-dimensional vector, and the positive real constants $\lambda_{\min}$ and $\lambda_{\max}$ represent the minimum and maximum eigen values of the matrix M(q) .

When Equation 51 is substituted, Equation 49 becomes

$$\dot{V}=e_{v}^{T}\delta -e_{v}^{T}K_{v}e_{v}.$$ (52)

The property Equation 50 yields

$$\dot{V}\leq -\gamma V+\sqrt{\frac{2V}{\lambda_{\min}}}|\delta |,\;\gamma =\frac{2K_{v}}{\lambda_{\max}}.$$ (53)

Based on the analysis in Sadegh and Horowitz (1990), the analysis is concluded as follows.

After division of Equation 53 by $V^{0.5}\neq 0$ , we get

$$\frac{d}{dt}\left(V^{0.5}\right)+0.5\gamma V^{0.5}\leq \sqrt{\frac{2}{\lambda_{\min}}}|\delta |,$$ (54)

Multiplying Equation 54 by $e^{-0.5\gamma }$ and performing integration yields

$$V^{0.5}\leq e^{-0.5\gamma t}V^{0.5}\left(0,e_{v}\left(0\right)\right)+V_{c},,$$ (55)

with

$$V_{c}=\frac{1}{\sqrt{2\lambda_{\min}}}\int_{0}^{t}e^{-0.5\gamma \left(t-\iota \right)}|\delta \left(\iota \right)|d\iota ,$$ (56)

where ι is a dummy variable representing time in the integral. It ranges from 0 to t (current time).

$V_{c}$ can be rewritten in the form

$$V_{c}=\frac{1}{\sqrt{2\lambda_{\min}}}\left(e^{-0.5\gamma t}*|\delta \left(t\right)|\right).$$ (57)

Applying the $\|{.\|}_{p}$ , where p is the norm order, leads to

$$\|V_{c}{\|}_{p}=\frac{1}{\sqrt{2\lambda_{\min}}}\|e^{-0.5\gamma t}*\delta \left(t\right){\|}_{p}.$$ (58)

Hence

$${\left\|V_{c}\right\|}_{p}=\frac{1}{\sqrt{2\lambda_{\min}}}{\left\|e^{-0.5\gamma t}\right\|}_{1}{\left\|\delta \left(t\right)\right\|}_{p}.$$ (59)

Since ${\left\|e^{-0.5\gamma t}\right\|}_{1}$ = 0.5γ , it follows that

$${\left\|V_{c}\right\|}_{p}=0.5\gamma \frac{1}{\sqrt{2\lambda_{\min}}}{\left\|\delta \left(t\right)\right\|}_{p}.$$ (60)

Consequently, Equation 55 becomes

$${\left\|V^{0.5}\right\|}_{p}\leq {\left\|e^{-0.5\gamma t}V^{0.5}\left(0,e_{v}\left(0\right)\right)\right\|}_{p}+0.5\gamma \frac{1}{\sqrt{2\lambda_{\min}}}{\left\|\delta \left(t\right)\right\|}_{p}.$$ (61)

Through simplification, Equation 61 becomes

$${\left\|e_{v}\right\|}_{p}\leq \frac{1}{\gamma }+\sqrt{\frac{2}{\lambda_{\min}}}{\left(\frac{2}{p\gamma }\right)}^{\frac{1}{p}}\sqrt{V\left(0,e_{v}\left(0\right)\right)}{\left\|\delta \right\|}_{p}.$$ (62)

Let $L_{p}$ refers to the space of signals with finite ${\left\|.\right\|}_{p}$ . Therefore, the error dynamics is $L_{p}$ input/output stable with respect to the pair ( δ , $e_{v}$ ) for all p∈[1,∞] with the assumption that the system states, q and $\dot{q}$ , are bounded.

Lemma 1.

The fuzzy system’s global asymptotic stability is guaranteed, supported by a Lyapunov function proof in Tanaka and Wang (2004).

Lemma 2. (Ioannou and Sun, 2012). Let

$$e=H_{\mathrm{FLC}}\;e_{v},$$ (63)

where $H_{\mathrm{FLC}}$ is exponentially stable. Then $e_{v}\in L_{p}$ implies that $e\in L_{p}$ and $\dot{e}\in L_{p}$ .

## 6 Simulation results

In this section, MATLAB/SIMULINK is utilized to simulate the previously suggested control technique for managing the aerial manipulation system under consideration.

### 6.1 Simulation environment

**FIGURE 6:**
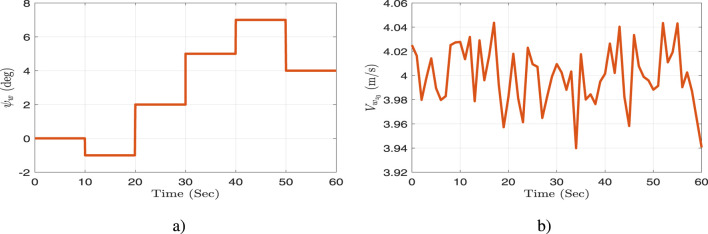
Profile of the wind: **(a)** angle, and **(b)** speed.

To ensure a realistic simulation, we have established the following setup and made specific assumptions:

• Our model is based on real data obtained from experimental tests Fanni and Khalifa (2017). The identified parameters are provided in Table 2.

• We access to the quadrotor’s linear and angular positions, as well as its velocities, at a 1 KHz sampling rate. Similarly, the manipulator joints’ positions and velocities are accessible at a 1 KHz sampling rate.

• To account for real-world conditions, we introduce measurement noise. Specifically, we add normally distributed noise to the measured signals, with a mean $10^{-3}$ and $5\times 10^{-3}$ standard deviation.

• Our controller computes outputs at a 1 KHz sampling rate.

• To assess robustness against model uncertainties, we introduce a step disturbance in the control matrix N (representing actuators’ losses) at 15 s. The disturbance assumes that the elements are 90% of their true values (i.e., a 10% error).

• A time-varying wind disturbance has been added. Figure 6a depicts a simulation of the wind angle profile, $\psi_{w}$ . Figure 6b shows that the wind speed $V_{w_{z_{0}}}$ has two components: a constant portion and a random variable portion to imitate gust effects (sudden and unpredictable variations in wind speed).

• Finally, the end-effector is tasked with picking up a 150 g payload at 15 s and releasing it at 55 s.

**TABLE 2:** Parameters of the new aerial manipulation system.

Par	Value	Unit	Par	Value	Unit
m	1	kg	$L_{2}$	$85\times 10^{-3}$	m
d	$223.5\times 10^{-3}$	m	$m_{0}$	$30\times 10^{-3}$	kg
$I_{x}$	$13.215\times 10^{-3}$	N.m.s^2^	$m_{1}$	$55\times 10^{-3}$	kg
$I_{y}$	$12.522\times 10^{-3}$	N.m.s^2^	$m_{2}$	$112\times 10^{-3}$	kg
$I_{z}$	$23.527\times 10^{-3}$	N.m.s^2^	$I_{r}$	$33.216\times 10^{-6}$	N.m.s^2^
$L_{0}$	$30\times 10^{-3}$	m	$L_{1}$	$70\times 10^{-3}$	m
$K_{F_{1}}$	$1.667\times 10^{-5}$	kg.m.rad^-2^	$K_{F_{2}}$	$1.285\times 10^{-5}$	kg.m.rad^-2^
$K_{F_{3}}$	$1.711\times 10^{-5}$	kg.m.rad^-2^	$K_{F_{4}}$	$1.556\times 10^{-5}$	kg.m.rad^-2^
$K_{M_{1}}$	$3.965\times 10^{-7}$	kg.m^2^.rad^-2^	$K_{M_{2}}$	$2.847\times 10^{-7}$	kg.m^2^.rad^-2^
$K_{M_{3}}$	$4.404\times 10^{-7}$	kg.m^2^.rad^-2^	$K_{M_{4}}$	$3.170\times 10^{-7}$	kg.m^2^.rad^-2^

### 6.2 Results and discussion

**FIGURE 7:**
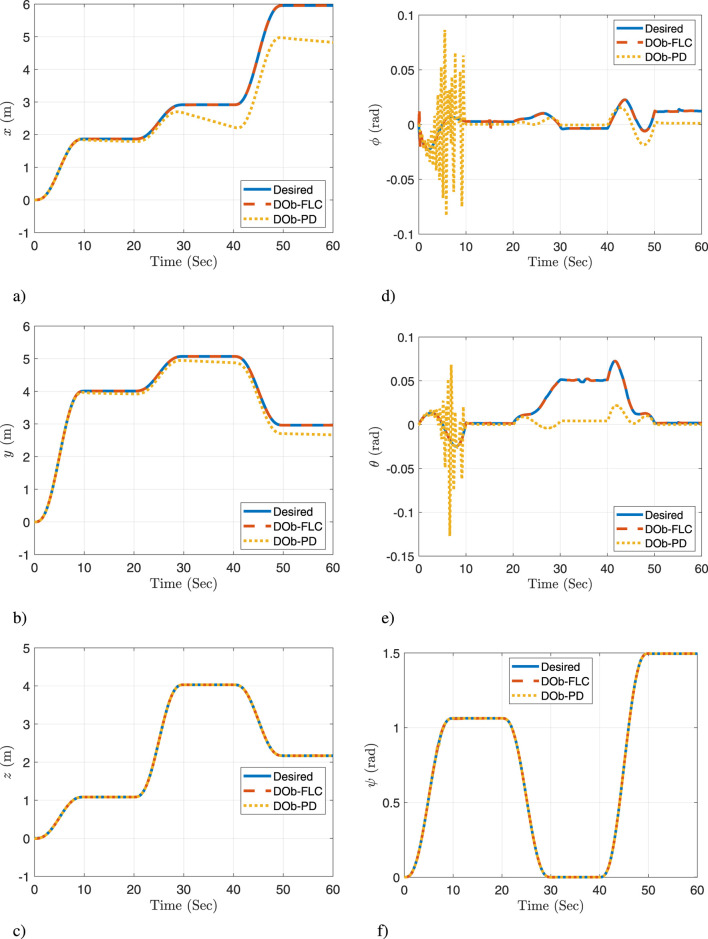
The actual response for the quadrotor space: **(a)** x , **(b)** y , **(c)** z , **(d)** ϕ , **(e)** θ , and **(f)** ψ

**FIGURE 8:**
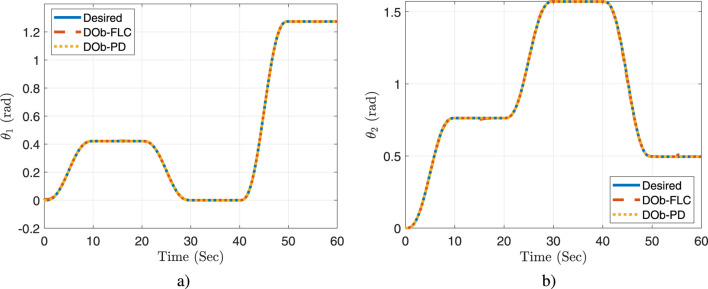
The actual response of the manipulator’s joints: **(a)** $\theta_{1}$ , and **(b)** $\theta_{2}$

A comparison of the proposed controller (DOb-FLC) to a DOb-PD controller is achieved. For the conducted simulation experiment, the actual response of the quadrotor space coordinate [x,y,z,ϕ,θ,ψ] is illustrated in Figure 7. While the actual response of the manipulator’s joints space coordinate $\left[\theta_{1},\theta_{2}\right]$ is presented in Figure 8. These figures demonstrate the feasibility of the new aerial manipulation system. Furthermore, they show that the quadrotor/joint space trajectories remain within the joints’ limits and do not breach the quadrotor/joints motion constraints. Also, they prove that the proposed controller is capable of effectively tracking the desired trajectories and rapidly correcting errors. The DOb-PD controller, on the other hand, has a steady state error and a limited ability to perform good trajectory tracking, particularly when faced with practical challenges. Thus, these results show that the proposed controller outperforms the DOb-PD controller’s response.

**FIGURE 9:**
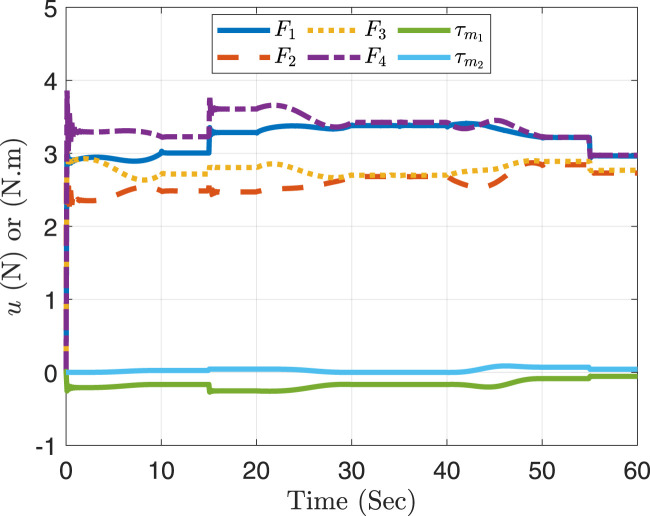
The required control efforts in case of DOb-FLC.

Additionally, Figure 9 demonstrates that the necessary efforts u from the actuators in the case of DOb-FLC, including the motor torque for each manipulator joint and the thrust force needed from each rotor, remain within the permissible range. The identification process determined that each rotor produces a maximum thrust force of 6 N. According to the motors’ data sheet, the motor for joint 1 has a permissible input torque of 0.7 N.m, while joint 2 has a permissible input torque of 0.4 N.m. Consequently, it can be argued that the desired control goals are accomplished through the implementation of this motion control strategy.

**FIGURE 10:**
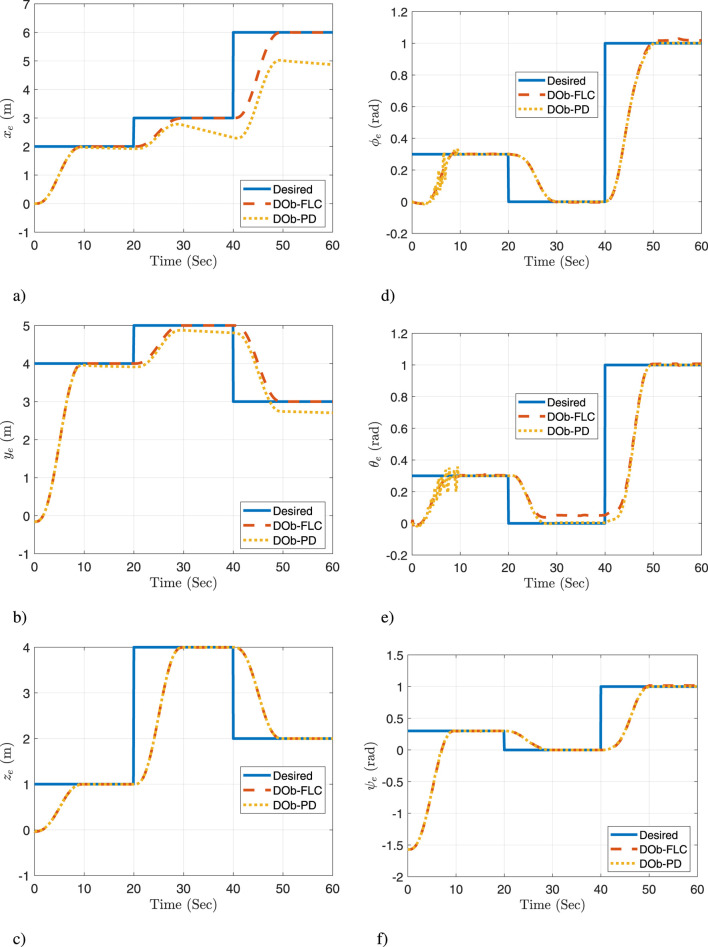
The actual response for the task space: **(a)** $x_{e}$ , **(b)** $y_{e}$ , **(c)** $z_{e}$ , **(d)** $\phi_{e}$ , **(e)** $\theta_{e}$ , and **(f)** $\psi_{e}$

Figure 10 illustrates the system’s response in the task space, where forward kinematics is utilized to compute the end effector’s actual position and orientation. This figure highlights the proposed method’s capability to precisely follow the desired 6-DOF end-effector trajectories, even when faced with practical challenges such as measurement noise, wind disturbances, and payload pickup or release. On the other hand, the DOb-PD controller has a steady state error and is less capable of performing good trajectory tracking, especially when faced with practical challenges. Therefore, it can be concluded that the proposed motion control approach is successful in meeting the control objectives.

### 6.3 Limitations and future extensions

The current study has some limitations that should be acknowledged. First, while the controller is designed for joint-space operation, most practical applications require accurate trajectory tracking in task space (rather than point-to-point) to ensure effective environmental interaction. Second, the control strategy is based on precise onboard positioning systems. Third, while the DOb assures bounded estimating error, this error does not equal zero, which may have an influence on performance in challenging environments that involve significant disturbances.

To address these limitations and expand upon this work, several promising research directions emerge. Future research should focus on experimental validation to evaluate the controller’s performance under realistic situations. The development of task-space control techniques, particularly sensor-based systems for direct environmental interaction, should be a priority. The disturbance estimating technique might be improved with algorithm enhancements targeted at either reaching zero estimate error or drastically reducing its bounds. These extensions would significantly improve the system’s practical applicability and performance.

## 7 Conclusion

For a quadrotor manipulation system, this paper investigates the challenge of more efficient and reliable robust linearization and control. The description of a new aerial manipulation system, consisting of a quadrotor vehicle and a 2-DOF manipulator that has a unique topology, is introduced. This unique topology enables the system to achieve 6-DOF trajectory tracking with a minimal number of actuators. Kinematics and dynamics analysis are investigated in detail. A modified DOb is utilized to ensure robust response by mitigating disturbances, noise in measurements, and mismatches between the actual plant and its model. In contrast to traditional approaches, the DOb uses measurement data from the encoders and IMU to estimate disturbances. Following this, the outer loop implements a fuzzy logic controller to attain the desired control objectives and closed-loop performance with minimal computational load. Stability analysis of the proposed controller is presented. The suggested control system was carefully tested using MATLAB/SIMULINK simulations. A comparison of the proposed controller to a DOb-PD controller is provided. The results show that the proposed controller outperforms the DOb-PD controller’s response. The results indicate precise trajectory tracking across all 6-DOF, even under diverse disturbances. The proposed controller maintains stable operation during payload handling while working within strict actuator limitations—specifically, rotor thrust remains below 6 N and joint torques remain below 0.7 N m (Joint 1) and 0.4 N m (Joint 2). These data verify the control strategy’s robustness and computational efficiency. In future work, the proposed system will undergo experimental testing.

## Data Availability

The raw data supporting the conclusions of this article will be made available by the authors, without undue reservation.

## Nomenclature

$\sum_{b}$: Body-fixed reference frame
∑: World-fixed inertial reference frame
$p_{b}$: Position relative to Σ , represented by ${\left[x\;y\;z\right]}^{T}$
$\Phi_{b}$: Orientation vector ${\left[\psi \;\theta \;\phi \right]}^{T}$ (ZYX yaw-pitch-roll angles)
$R_{b}$: Rotation matrix from body frame to world frame
$\sum_{e}$: Frame attached to manipulator’s end-effector
$p_{e}$: Position of $\Sigma_{e}$ relative to Σ
$p_{eb}^{b}$: Position of $\Sigma_{e}$ relative to $\Sigma_{b}$ , expressed in $\Sigma_{b}$
$R_{e}$: Rotation matrix of $\Sigma_{e}$
$R_{e}^{b}$: Rotation matrix specifying orientation of $\Sigma_{e}$ w.r.t $\Sigma_{b}$
$\chi_{e}$: Operational task coordinates
q: Vehicle/joint space coordinates
$\theta_{i}$: joint i angle of the manipulator (i=1,2)
$L_{0}$: Length of link attached to quadrotor (base link)
$L_{1}$: Length of first manipulator link
$L_{2}$: Length of second manipulator link
m: Quadrotor’s total mass
$\Omega_{j}$: Angular velocity of rotor j (j=1,2,3,4)
$F_{j}$: Thrust force of rotor j
$M_{j}$: Drag moment of rotor j
$K_{f_{j}}$: Thrust coefficient for rotor j
$K_{m_{j}}$: Drag coefficient for rotor j
T: Total thrust
d: Perpendicular distance from center of mass to rotor axis
$\bar{\Omega }$: Rotor speed vector
$I_{r}$: Inertia of the rotor
$I_{x},I_{y},I_{z}$: Quadrotor moments of inertia about body axes
M(q): System inertia matrix
$C\left(q,\dot{q}\right)$: Matrix of Coriolis and centrifugal terms
G(q): Vector of gravity terms
$d_{ex}$: Vector of external disturbances
u: Actuator input vector
B: Input matrix
N: Control matrix
H: Transformation matrix for body input forces to Σ
$\tau_{m_{i}}$: Torque on manipulator joint i (i=1,2)
$F_{m,q_{x}}$: Manipulator force on quadrotor along x
$F_{m,q_{y}}$: Manipulator force on quadrotor along y
$F_{m,q_{z}}$: Manipulator force on quadrotor along z
$M_{m,q_{\phi }}^{b}$: Manipulator moment about $x_{b}$
$M_{m,q_{\theta }}^{b}$: Manipulator moment about $y_{b}$
$M_{m,q_{\psi }}^{b}$: Manipulator moment about $z_{b}$
$\tau^{\mathrm{dis}}$: System disturbances
${\hat{\tau }}^{\mathrm{dis}}$: System disturbances
${\hat{\tau }}^{\mathrm{dis}}$: System estimated disturbances
$M_{n}$: Nominal inertia matrix
Q(s): DOb low-pass filter matrix
P: Bandwidth matrix
$g_{i}$: Low-pass filter bandwidth of $i^{th}$ variable
$\psi_{w}$: Wind angle
$V_{wz}$: Wind speed at altitude z
$F_{w}$: Wind disturbance force
$A_{e}$: Quadrotor’s effective impact area
e: Error signal in fuzzy controller
de: Rate of change of the error
